# The Possible Role of Naringenin in the Prevention of Alcohol-Induced Neurochemical and Neurobehavioral Deficits

**DOI:** 10.1007/s11064-022-03775-x

**Published:** 2022-10-15

**Authors:** Nema A. Soliman, Muhammad T. Abdel Ghafar, Norhan A. AbuoHashish, Marwa A. Ibrahim, Asmaa M. Eid, Rehab M. El-Gohary, Rehab E. Abo El gheit, Amira M. Elshamy

**Affiliations:** 1 Department of Medical Biochemistry, Faculty of Medicine, Tanta University, Tanta, Egypt; 2 Department of Clinical Pathology, Faculty of Medicine, Tanta University, Aljaysh St, Medical Campus, Tanta, 31511 Egypt; 3 Department of Pharmacology, Faculty of Medicine, Tanta University, Tanta, Egypt; 4 Department of Histology, Faculty of Medicine, Tanta University, Tanta, Egypt; 5 Department of Pathology, Faculty of Medicine, Tanta University, Tanta, Egypt; 6 Department of Physiology Faculty of Medicine, Tanta University, Tanta, Egypt

**Keywords:** Neurodegeneration, Alcohol, Naringenin, Oxidative stress, Neuroprotection

## Abstract

Chronic alcohol consumption is associated with progressive/irreversible neurodegeneration. However, there is not a clear understanding of its discrete pathophysiology or therapeutic intervention. The present study aimed to investigate the protective effect of the natural citrus flavonoid, naringenin (NAG), against alcohol-induced neurodegeneration in the brain cerebral cortex. Thirty-two male albino rats were randomly divided into four equal groups (eight rats each): control group (I); NAG-treated group (II); alcohol-intoxicated group (III) and alcohol + NAG co-treated group (IV). Brain nuclear factor erythroid 2-related factor 2 and receptor-interacting protein kinase 3 expression were assessed by real-time polymerase chain reaction. NAD(P)H quinone oxidoreductase 1 activity and malondialdehyde, reduced glutathione, mixed lineage kinase-like protein, phosphorylated glycogen synthase kinase 3 beta, and ciliary neurotrophic factor levels were all measured biochemically. B-cell lymphoma 2 expression was assessed by immunohistochemistry. A histopathological examination and neurobehavioral tests were performed. The alcohol-treated group showed a significant increase in oxidative stress and necroptosis biomarkers with a significant reduction in neuroprotective proteins. NAG co-administration effectively ameliorated cognitive dysfunction with an apparent neuroprotective effect by targeting various signaling pathways, including nuclear factor erythroid 2-related factor/NAD(P)H quinone oxidoreductase 1, anti-oxidant capacity, attenuated necroptosis, and upregulated neuroprotective ciliary neurotrophic factor. The study findings suggest NAG as a possible management strategy for alcohol-induced neurodegeneration.

## Introduction

Alcoholism is a medical condition characterized by a wide range of symptoms that impair physical, mental, and social health [1]. Due to the high degree of similarity between human and animal brains, animal models can be used to conduct comprehensive investigations into alcohol-induced chemical, physiological, and genetic alterations in the brain in terms of cell apoptosis, proliferation, and brain anatomical and functional alterations [1]. Alcohol is a well-known neurotoxic agent that is widely consumed all over the world. It can cause serious adverse effects, such as progressive damage to multiple tissues, including the brain [2].

Alcohol has been shown to induce brain cell damage, as evidenced by numerous animal experiments, with subsequent loss of brain cell structure or function (i.e., neurodegeneration) of various areas within the brain, which is comparable to that induced in human alcoholics. In addition, animal studies have revealed that alcohol inhibits neurogenesis and other brain-cell genesis, resulting in alcoholic neurodegeneration [2]. Alcohol-induced neurodegeneration has intricate mechanisms that are similar in many aspects and modulators to those of other neurodegenerative disorders. The most important mechanisms of alcohol-induced neurodegeneration are necroptosis and apoptosis, which both play central roles in the pathogenesis of neurodegenerative disorders [2]. Apoptosis is a caspase-mediated process required for neural development and is controlled by members of the anti-and pro-apoptotic B-cell lymphoma-2 (BCL-2) protein family [3]. Necroptosis, a genetically designed and controlled form of necrosis, and its signaling pathway have emerged as an adaptive and pathological component in the pathophysiology of several neurodegenerative disorders [4]. Necroptosis can be induced by death receptor activation, which subsequently activates receptor-interacting protein kinase 3 (RIPK3) and mixed lineage kinase-like protein (MLKL). In this instance, MLKL phosphorylation by RIPK3 eventually causes necroptosis via disrupting the plasma membrane, cell lysis, and neuroinflammation [5]. Neurodegenerative disorders could be alleviated in vitro and in vivo by inhibiting necroptotic signaling, implying a promising treatment strategy for neurodegenerative disorders [6].

Glycogen synthase kinase 3 (GSK-3) is implicated in multiple signaling cascades that regulate cellular metabolism, differentiation, and immunity, as well as cell death and survival [7]. It is a multifunctional Ser/Thr kinase that is negatively regulated by phosphorylation. Two GSK-3 isoforms, GSK-3α and GSK-3β, have been identified, with GSK-3β playing a significant role in the pathogenesis of many disorders and serving as a crucial molecular therapeutic target in neurodegenerative disorders [8]. In neurodegenerative animal models, GSK-3 has recently been identified as a major regulator of peripheral inflammatory responses, with a high capacity to promote the production of several cytokines [9, 10]. The role of oxidative stress in neurological dysfunction and diseases has been demonstrated to be an attractive therapeutic target. GSK-3β appears to modulate the cellular response to oxidative stress, which is a characteristic feature of many neurological diseases, by interacting with the nuclear factor erythroid 2-related factor 2 [11].

Nuclear factor erythroid 2-related factor 2 (Nrf2) is a novel regulator that mediates resistance to various cellular oxidants. It upregulates the expression of various antioxidant response element-dependent genes, thereby regulating the physiological and pathological effects of oxidants [12].

Naringenin (NAG), a dietary flavonoid, is abundant in citrus fruits such as bergamot, orange, lemon, and grapefruit. It has numerous biological activities, including antitumor, anti-adipogenic, antioxidant, anti-inflammatory, antiviral, antibacterial, and cardioprotective effects [13]. The current study aimed to investigate the protective effect of NAG against alcohol-induced neurodegeneration in the cerebral cortex, as well as the potential role of alcohol-induced oxidative stress and necroptosis signaling pathways in the pathogenesis of neurodegeneration and the potential therapeutic intervention by the citrus flavonoid NAG.

## Materials and Methods

### Drugs and Chemicals

NAG (< 95%) and ethanol (99.8%) were supplied by Sigma Chemicals Co. (St. Louis, Missouri, United States, catalog no. N5893 and 64-17-5, respectively).

### Animals and Experimental Design

In this study, 32 male Swiss albino rats (35 days old, weighing 160–190 g at the start of the experiment) were kept in well-ventilated wire mesh cages at normal temperature (25 ± 2 °C) and humidity (40–45%) in a light-controlled room with 12-h dark/light cycles and unrestricted access to food and water. After 2 weeks of acclimation, rats were divided into four equal groups of eight rats each, as follows: Group I (control group) was given 200 µl of normal saline orally; Group II (NAG-treated group) was given 50 mg/kg/day of NAG [14], along with 200 µl of normal saline once daily; Group III (alcohol-intoxicated group) was given 6.5 g/kg/day of 22.5% (w/v) ethanol once daily [15]; and Group IV (NAG co-treated group) was given 6.5 g/kg/day of 22.5% (w/v) ethanol, along with 50 mg/kg/day of NAG once daily. All treatments were given orally by gavage for 55 days. Throughout the experimental period, rats were monitored on a daily basis, and their survival rates in all groups were carefully assessed. There were no deaths in any of the experimental groups. At the end of the experiment, body weights were measured for all groups studied. This research adhered to the guidelines of the National Institutes of Health for the care and use of laboratory animals (NIH Publications No. 8023, revised 1996) and was approved by the Research Ethics Committee, Faculty of Medicine, Tanta University, Egypt (Approval No. 33796/4/20).

### Assessment of Neurobehavioral Impairments

The behavioral tests were performed 24 h after the last day of the experiment (Day 55) by an experienced experimenter blinded to the experimental groups. Prior to the neurobehavioral tests, animals were brought to the assay room and acclimated for at least an hour.

#### Open Field Test

In a 5-min session test in an open-field environment, each animal’s spontaneous locomotor activity was assessed. The arena is made of wood coated with impermeable Formica (100 × 100 × 40 cm), with a white floor divided into 25 squares of 20 × 20 cm each by black lines, and white walls. Each rat was placed in the open field center and then was given one minute to explore its surroundings. The number of squares crossed by the rat was then recorded for 5 min using a video camera [15].

#### Inclined Plane Test

After the open field test, an inclined plane test was carried out to assess the animal’s capacity to preserve postural permanency. On an inclined plane stage (50 × 75 cm) with a grooved 1-mm thick rubber surface, rats were positioned with their heads down. The angle was adjusted in 5° increments until the rat could no longer maintain its position for 5 s. Each rat was evaluated in five consecutive trials separated by a 60-s interval. To determine the extreme learning, the last angle (in degrees) at which the rat could maintain its position for 5 s was recorded [16].

### Cerebral Cortex Tissue Sampling

Immediately following neurobehavioral testing, rats were sacrificed under pentobarbital sodium anesthesia. Each rat’s whole brain was dissected, and its volume was measured using a Vernier caliper to determine brain dimensions such as anterior-posterior brain length [mm] and lateral brain width [mm], and the volume was calculated using the formula: *Volume (V) (mm*^*3*^*) = 0.5 (length × width*^*2*^*)* [17, 18]. Brain was then separated rapidly on ice with a surgical blade before being weighed. The right lobe of the cerebral cortex was rinsed with ice-cold saline and then cut into pieces. One piece was homogenized in an ice cold 20 mM Tris–HCl buffer (pH 7.4) using a Potter–Elvenhjem tissue homogenizer at a fixed concentration of 10 mL/g tissue. The samples were processed at speed 4 for three cycles of 15 s each with a dwell time of 30 s. The homogenate was then centrifuged for 40 min at 14.000 rpm and 4 °C [19]. The obtained cytosolic supernatant was kept at − 80 °C for further biochemical analysis. Another was kept at − 80 °C for molecular testing. Protein content was assessed according to Lowry et al. [20].

### Immunoassay of Mixed Lineage Kinase Domain Like Protein (MLKL), Phosphorylated (p) GSK-3β, and Ciliary Neurotrophic Factor (CNTF)

The MLKL, phosphorylated (p) GSK-3β, and CNTF levels in brain tissue were assayed using enzyme-linked immunosorbent assay (ELISA) kits (Cloud-Clone Corp, Wuhan, China, catalog no. SER645Ra; MyBiosource, Inc., San Diego, USA, catalog no. MBS730623; and RayBiotech Inc., Georgia, USA, catalog no. ELR-CNTF, respectively) according to the manufacturer’s protocol. The absorbance was measured at 450 nm with 570 nm as the correction wavelength using a microplate reader (Stat Fax®2100, Fisher Bioblock Scientific, France).

### Assessment of Oxidant/Antioxidant Status Biomarkers

Malondialdehyde (MDA) levels in brain tissue were measured as previously described [21]. In brief, thiobarbituric acid reactive substances were measured at 532 nm and calculated using an extinction coefficient of malondialdehyde-thiobarbituric acid (MDA‐TBA) complex of 1.56 × 10^5^ M^−1^ cm^−1^, with the results expressed as nmol/gm wet tissue. In addition, reduced glutathione (GSH) levels were calorimetrically measured at 405 nm using commercial kits supplied by Biodiagnostic, Giza, Egypt. In brief, GSH reduces 2-nitrobenzoic acid (DTNB), resulting a yellow reduced chromogen that is directly proportional to GSH concentration, which is expressed as mg/gm wet tissue. Furthermore, the NAD(P)H quinoneoxidoreductas1(NQO1) activity in brain tissue was assessed colorimetrically as previously described [22] by oxidizing NADPH to NADP + using 2,6-Dichlorophenolindophenol as a substrate and measuring the disappearance of NADPH at 600 nm over one minute. All colorimetric assays were performed using a semi-automatic BTS 350 chemistry analyzer (Biosystems, Spain).

### Relative *RIPK3* and *Nrf2* Genes Expression Assay by Quantitative Real Time PCR

Frozen brain tissues were processed, and total RNA was extracted using the Qiagen RNeasy Total RNA isolation kit (Qiagen, Hiden, Germany) according to the manufacturer’s instructions. RNA was reverse transcribed to cDNA using the SuperScript ® III FirstStrand Synthesis System for RT-PCR kit (Life Technologies). PCR was performed using Power SYBR Green PCR Master Mix (Life Technologies). *RIPK3* and *Nrf2 mRNA* expression levels were calculated relative to the housekeeping gene, glyceraldehyde 3-phosphate dehydrogenase (GAPDH). The Primer3 software (http://bioinfo.ut.ee/primer3/) was used to design the primers as follows: rat *RIPK3* F: 5′‐CAGTGTTGGCTGGAAGAGAA‐3′; R: 5′‐AGGCTCAGAACTCCAGCAAT‐3′ (GenBank Accession No. NM_139342.1); rat *Nrf2* F: 5′‐TAGCAGAGCCCAGTGGCGGT-3′; R: 5′‐TGCTCTGGGGATGCTCGGCT-3′ (GenBank Accession No. NM_031789.1); and rat *GAPDH* F: 5′‐GGTGAAGTTCGGAGTCAACGGA‐3′; R: 5′‐GAGGGATCTCGCTCCTGGAAGA‐3′ (GenBank Accession No. NM_017008). The following cycling profile was used: Pre-denaturation at 95 °C for 10 min, followed by 40 cycles of 95 °C for 15 s, 65 °C for 1 min, and 72 °C for 1 min. The relative gene expression was calculated based on the comparative cycle thresholds of the target and reference genes by RotorGene Q6plex and its associated software (Qiagen, Valencia, CA, USA).

### Histopathological Examination

For the histological study, formalin-fixed brain specimens from the left cerebral cortex of the frontal lobe of each rat brain were dissected and immediately immersed in 10% neutral buffered formalin. The formalin-fixed brain specimens were washed, dehydrated, cleared, and embedded in paraffin. The 5 μm thick sections were stained with hematoxylin and eosin stain (H&E) [23].

### Immunohistochemistry for B-Cell Lymphoma-2 (BCL-2) Detection

For immunohistochemical (IHC) staining, 5 μm thick sections were deparaffinized, rehydrated, and washed with phosphate-buffered saline (PBS). Endogenous peroxidase was neutralized by 10% hydrogen peroxide. Sections were treated with a rabbit polyclonal BCL-2 primary antibody (ab59348; Abcam, Cambridge, Massachusetts, USA) and incubated overnight at 4 °C in a humid chamber. Afterward, goat anti-rabbit peroxidase-conjugated secondary antibody was applied to tissue sections, incubated for 60 min at room temperature, and then rinsed with PBS. The immunoreactivity was visualized by applying streptavidin peroxidase substrate (3,3-diaminobenzidine–hydrogen peroxide) as a chromogen and counterstained with Mayer’s hematoxylin. Human colon carcinoma tissue served as the positive control, while the primary antibody was replaced by PBS as a negative control [24]. A cytoplasmic brownish coloration indicated a positive immunoreaction. For image acquisition, a Leica light microscope (DM500, Switzerland) coupled with a Leica digital camera (ICC50, Switzerland) was used. Morphometrical analysis was performed using “ImageJ” software (version 1.48v, National Institute of Health, Bethesda, Maryland, USA), with ten different non-overlapping randomly selected fields from each slide examined at a magnification of 400 to quantify the mean color intensity of BCL-2 positive IHC reaction.

### Statistical Analysis

All data were statistically analyzed and presented as mean and standard deviation (SD) using the Statistical Package for the Social Sciences (SPSS) software, version 21 (IBM, SPSS Inc., Armonk, NY, USA). The Graphpad prism software, version 9.3.1 (San Diego, CA, USA) was used to create graphs. All variables were checked using a two-way analysis of variance (ANOVA) test while considering the ethanol and NAG treatments, which revealed a significant interaction between the ethanol and NAG treatments, leading us to establish the four studied groups based on these two factors and compare them using one-way ANOVA, with a post hoc Tukey test used to assess the significance between two groups. A *p-*value of less than 0.05 was considered statistically significant.

## Results

### Effect of NAG Treatment on Body Weight and Brain Weight, Volume, and Total Protein Content

**Fig. 1:**
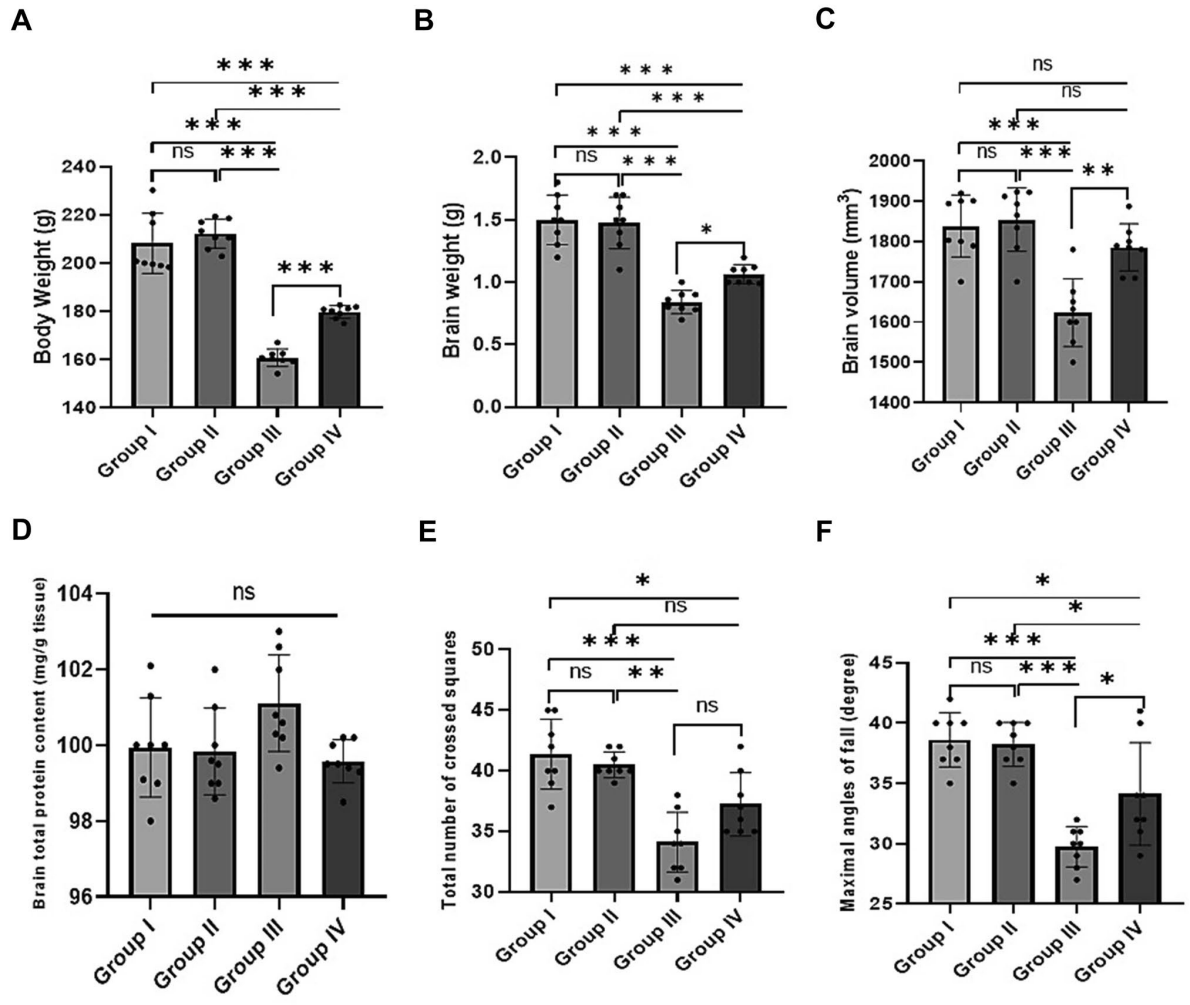
Effect of naringenin treatment on brain weight and neurobehavioral deficits (open field and inclined plane tests) in all studied groups. **A** Body weight. **B** Brain weight. **C** Brain volume. **D** Brain total protein content. **E** Total number of crossed squares. **F** Maximal angles of fall. The values are represented as the means and standard deviations of eight rats in each group. ns: non-significant, *p < 0.05, **p < 0.01, and ***p < 0.001

As shown in Fig. 1 A–C, alcohol-induced brain toxicity was clearly demonstrated, as lower trends in body weight and brain weight and volume were observed in Group III than in other groups (all *p* < 0.001). NAG co-treatment markedly preserved body weight and brain weight and volume, as evidenced by a significant increase in body weight and brain weight and volume of Group IV when compared to the alcohol-intoxicated group (*F* = 89.87, 63.6, and 15.7, respectively, all *p* < 0.001). The findings suggest a positive association between excessive alcohol consumption and catastrophic loss of brain tissue as well as decreased body weight, as alcohol may cause severe malnutrition because alcoholics typically consume 50% of their caloric intake from alcohol. Furthermore, there was a minor, non-significant variation in brain total protein content across all experimental groups (*F* = 3.01, *p* = 0.056, Fig. 1D).

### Effect of NAG Treatment on Ethanol-Induced Neurobehavioral Deficits

NAG co-treatment improved locomotor activity in the open arena as measured by an increase in the total number of squares crossed by treated rats, though this did not reach statistical significance when compared to the ethanol-treated group (*p =* 0.06) (Fig. 1E). The rat’s ability to maintain its posture during the inclined plane test is depicted in Fig. 1F. When compared to the alcohol-treated group, NAG co-treatment significantly increased the maximum angle score (*p* = 0.013). Accordingly, these findings suggest that NAG may provide neuroprotection against alcohol-induced neurobehavioral deficits.

### Effect of NAG on Brain Oxidant/Antioxidant Status Biomarkers

**Fig. 2:**
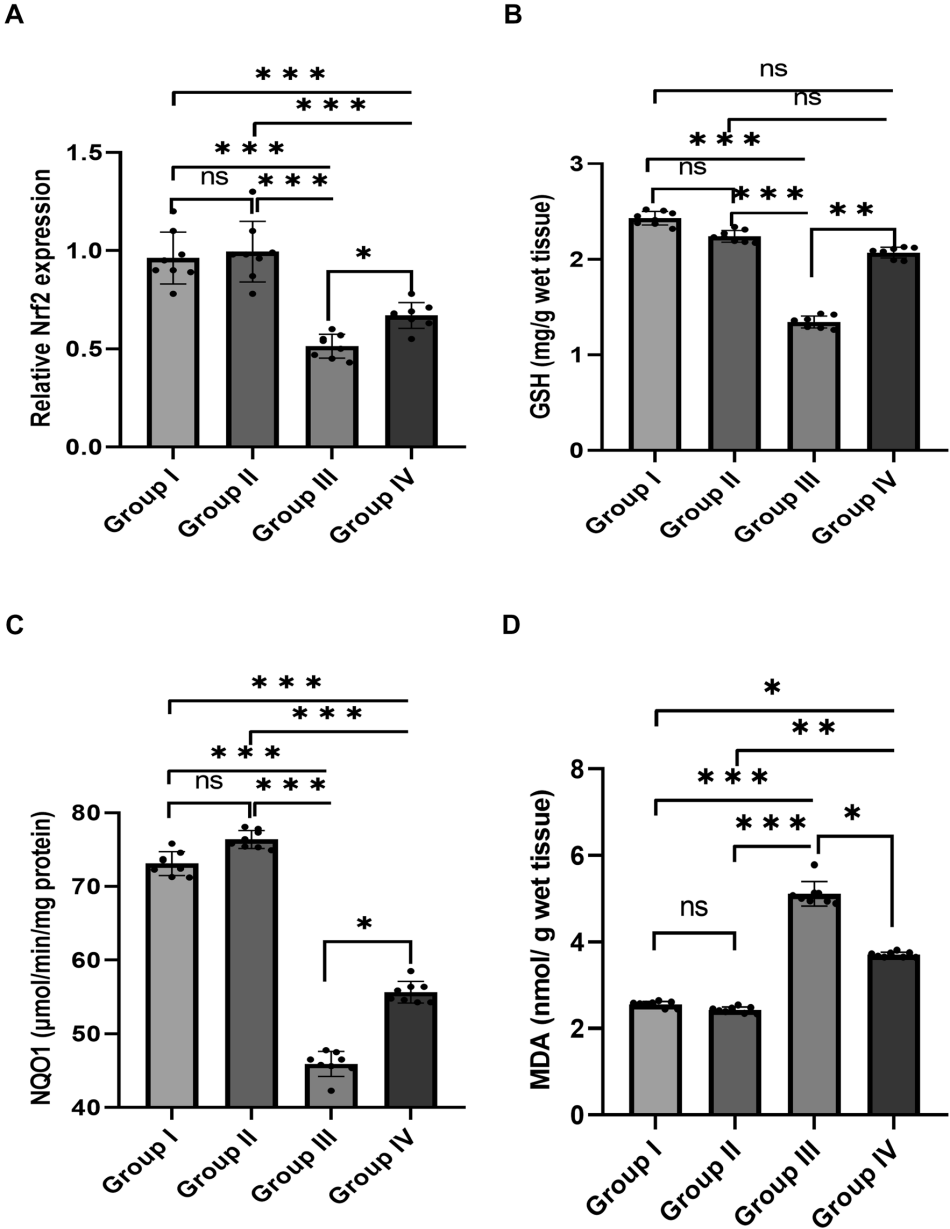
Effect of NAG on brain oxidant/antioxidant status biomarkers. **A** Relative *Nrf2* gene expression. **B** GSH level (mg/g wet tissue). **C** NQO1 (µmol/min/mg protein). **D** MDA level (nmol/ g wet tissue). The values are represented as the means and standard deviations of eight rats in each group. ns: non-significant, *p < 0.05, **p < 0.01, and ***p < 0.001

As illustrated in Fig. 2, NAG significantly alleviated ethanol-induced oxidative stress by improving antioxidant status and reducing oxidative stress-related biomarkers. The NAG co-treated group showed a significant increase in *Nrf2* mRNA gene expression (Fig. 2A), GSH level (Fig. 2B), and NQO1 activity (Fig. 2C) when compared to the ethanol-treated group (*p* = 0.042, *p* < 0.001, *p* < 0.001, respectively), while the MDA level (Fig. 2D) showed the reverse (*p* < 0.001), with significant differences in the aforementioned parameters among various groups (*F* = 35.03, 443.01, 735.61, and 529.9, respectively, all *p* < 0.001). These findings highlight the significance of NAG as an oxidative response stabilizer that plays an important role in the prevention of alcohol-related neurotoxicity.

### Effect of NAG on Brain Necroptosis Biomarkers

**Fig. 3:**
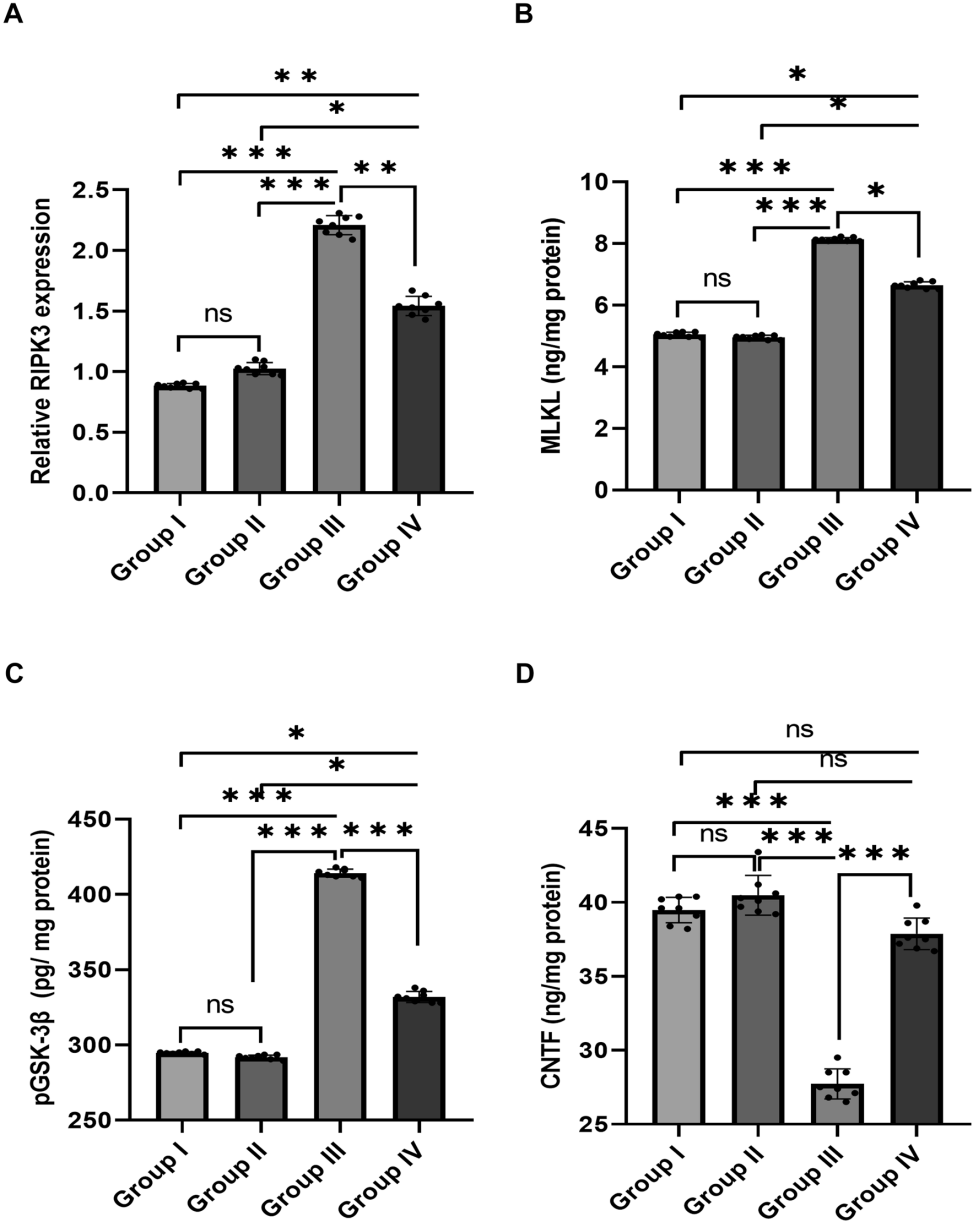
Effect of NAG on brain necroptosis and neuroprotection biomarkers. **A** Relative *RIPK3* gene expression. **B** MLKL (ng/mg protein). **C** pGSK-3β (pg/ mg protein). **D** CNTF (ng/mg protein). The values are represented as the means and standard deviations of eight rats in each group. ns: non-significant, *p < 0.05, **p < 0.01, and ***p < 0.001

*RIPK3* mRNA expression (Fig. 3A) and MLKL levels (Fig. 3B) were significantly higher in the alcohol-intoxicated group (III) than in the other groups. Notably, NAG co-treatment in Group IV resulted in a significant decrease in the aforementioned biomarkers (*F* = 755.3 and 2861.5, respectively, *p* < 0.001). These findings demonstrated that NAG co-administration enhanced all transformed factors and provided insight into a possible molecular mechanism underlying NAG suppression of necroptosis signaling.

### Effect of NAG on Brain Neuroprotective Biomarkers

The pGSK-3β levels (Fig. 3C) were significantly higher in the ethanol intoxicated group than in the other studied groups, while the CNTF levels (Fig. 3D) were decreased. NAG co-treatment resulted in a significant decrease in pGSK-3β and a significant increase in CNTF in Group IV (*F* = 4506.2 and 234.5, respectively, all *p* < 0.001), indicating NAG’s neuroprotective effect. These findings imply that NGN could be used as a therapeutic target in alcohol-induced neurodegenerative disorders.

### Histopathological and Immunohistochemical Evaluation

**Fig. 4:**
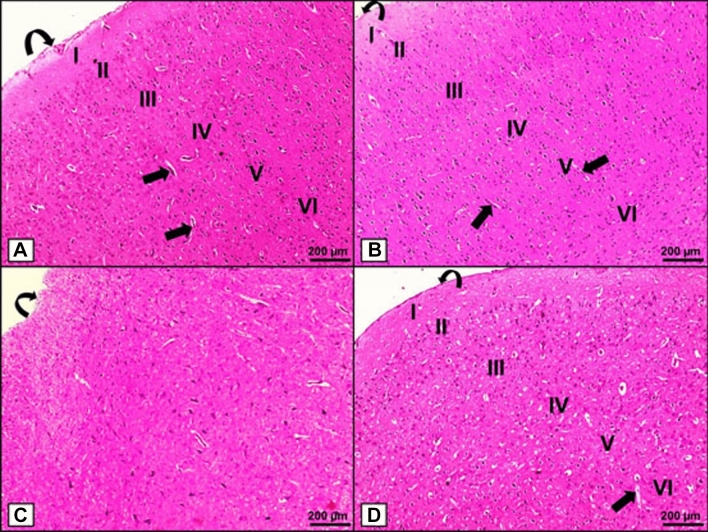
H & E histological staining (x100). **A**, **B** Groups I and II show the normal histoarchitecture of the cerebral cortex with an outer lining of pia mater (curved arrow), six underlying layers; outer molecular (I), external granular (II), external pyramidal (III), inner granular (IV), inner pyramidal (V), and polymorphic layer (VI), and numerous blood vessels (thick arrows). **C** Group III shows apparent linear disorientation of the cerebral cortical layers with thickened irregular pia mater lining (curved arrow). **D** Group IV shows an apparently normal histoarchitecture of the cerebral cortex with the proper linear arrangement, intact pia mater lining (curved arrow), and blood vessels (thick arrow). [Magnifications: (a-d) x100, scale bar = 200 μm]

H&E-stained sections of the cerebral cortex from Groups I and II revealed normal histoarchitecture with an outer lining of the pia mater and six underlying layers, starting with outer molecular, external granular, external pyramidal, inner granular, inner pyramidal, and ending with a polymorphic layer. Multiple blood vessels were detected in all layers (Fig. 4A, B). Sections from Group III, on the other hand, showed obvious linear disorientation of the cerebral cortical layers with thickened irregular pia mater lining (Fig. 4C). Meanwhile, H&E-stained sections from Group IV revealed an apparently normal histoarchitecture of the cerebral cortex with a proper linear arrangement and an intact pia mater lining (Fig. 4D).

**Fig. 5:**
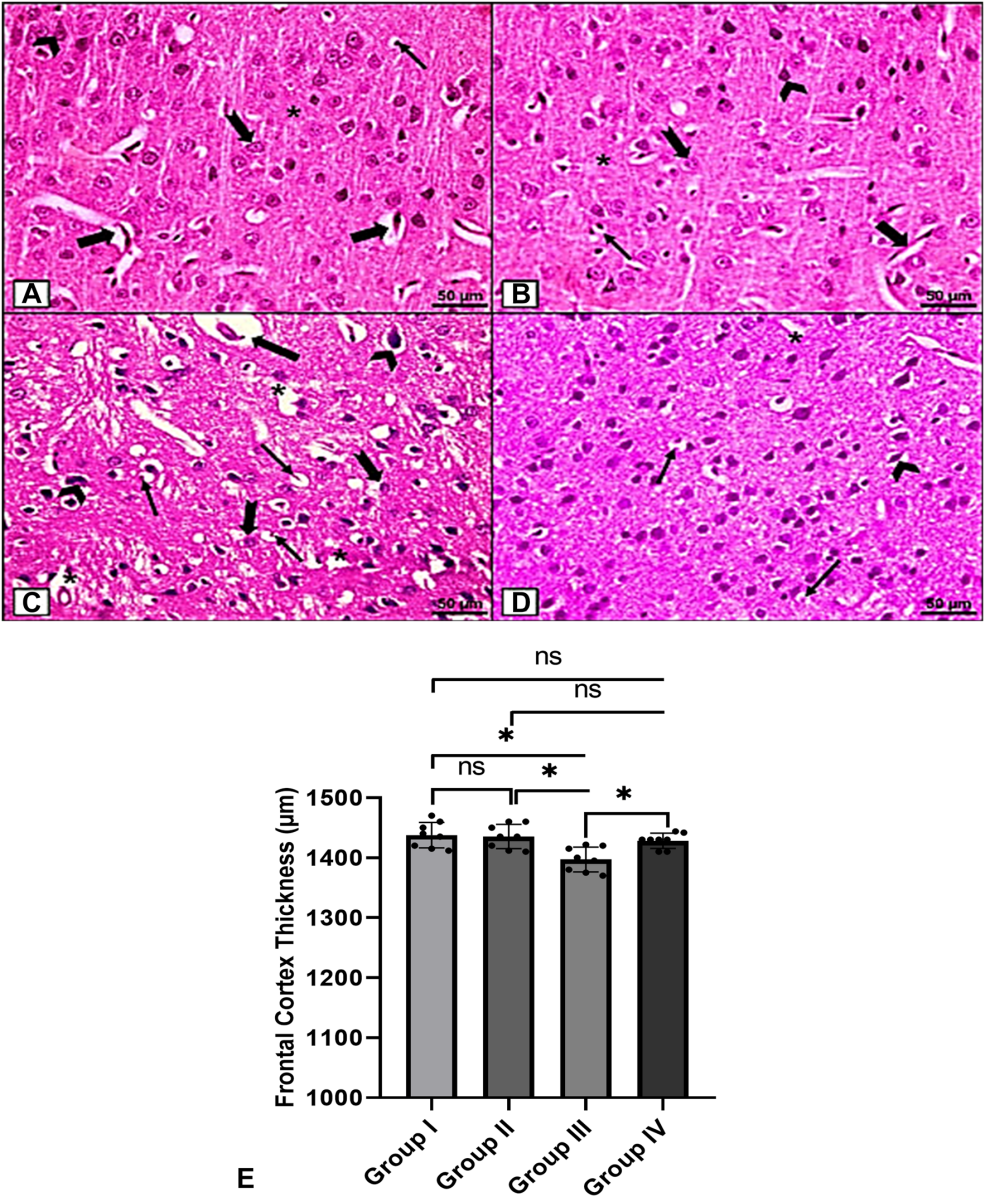
H & E histological staining (x400). **A**, **B** Groups I and II show pyramidal cells (arrowhead) with characteristic apical dendrites and vesicular nuclei, granule cells (notched arrow) with large open-face nuclei, glial cells (thin arrow), and blood vessels (thick arrows) embedded within the neuropil (asterisk). **C** Group III shows shrunken apoptotic pyramidal cells (arrowheads), vacuolated granular cells (notched arrows), some degenerated red neurons (thin arrows), blood vessels with dilated perivascular spaces (thick arrow), and multiple degenerated vacuoles in the neuropil (asterisks). **D** Group IV shows the intact morphology of most pyramidal cells, granular cells, neurons, and blood vessels, yet a few shrunken pyramidal cells (arrowhead), red neurons (thin arrow), and some vacuoles in the neuropil (asterisk) are detected. [Magnifications; (a-d) x400, scale bar = 50 μm]. **E** Morphometric analysis of the mean thickness of the frontal cortex. The values are represented as the means and standard deviations of eight rats in each group. ns: non-significant, *p < 0.05, **p < 0.01, and ***p < 0.001

H&E-stained sections of higher magnification from Groups I and II revealed pyramidal cells with characteristic apical dendrites, vesicular nuclei, granule cells with large open-face nuclei, glial cells, and blood vessels embedded within the neuropil (Fig. 5A, B). However, sections of Group III depicted shrunken apoptotic pyramidal cells, vacuolated granular cells, degenerated red neurons, blood vessels with dilated perivascular spaces, and multiple degenerated vacuoles in the neuropil (Fig. 5C). Sections of Group IV, on the other hand, revealed the intact morphology of most pyramidal cells, granular cells, and neurons, with the exception of a few shrunken pyramidal cells, red neurons, and some vacuoles in the neuropil (Fig. 5D). These findings suggest that NAG had a protective effect on the rat brain during degenerative structural events.

Morphometric analysis revealed that the mean thickness of the frontal cortex differed significantly among the studied groups (*F* = 6.67, *p* = 0.002), with Group III having a significantly lower mean thickness than the control group (*p* = 0.010) and Group IV having a significantly higher mean thickness than Group III (*p* = 0.022) (Fig. 5E).

**Fig. 6:**
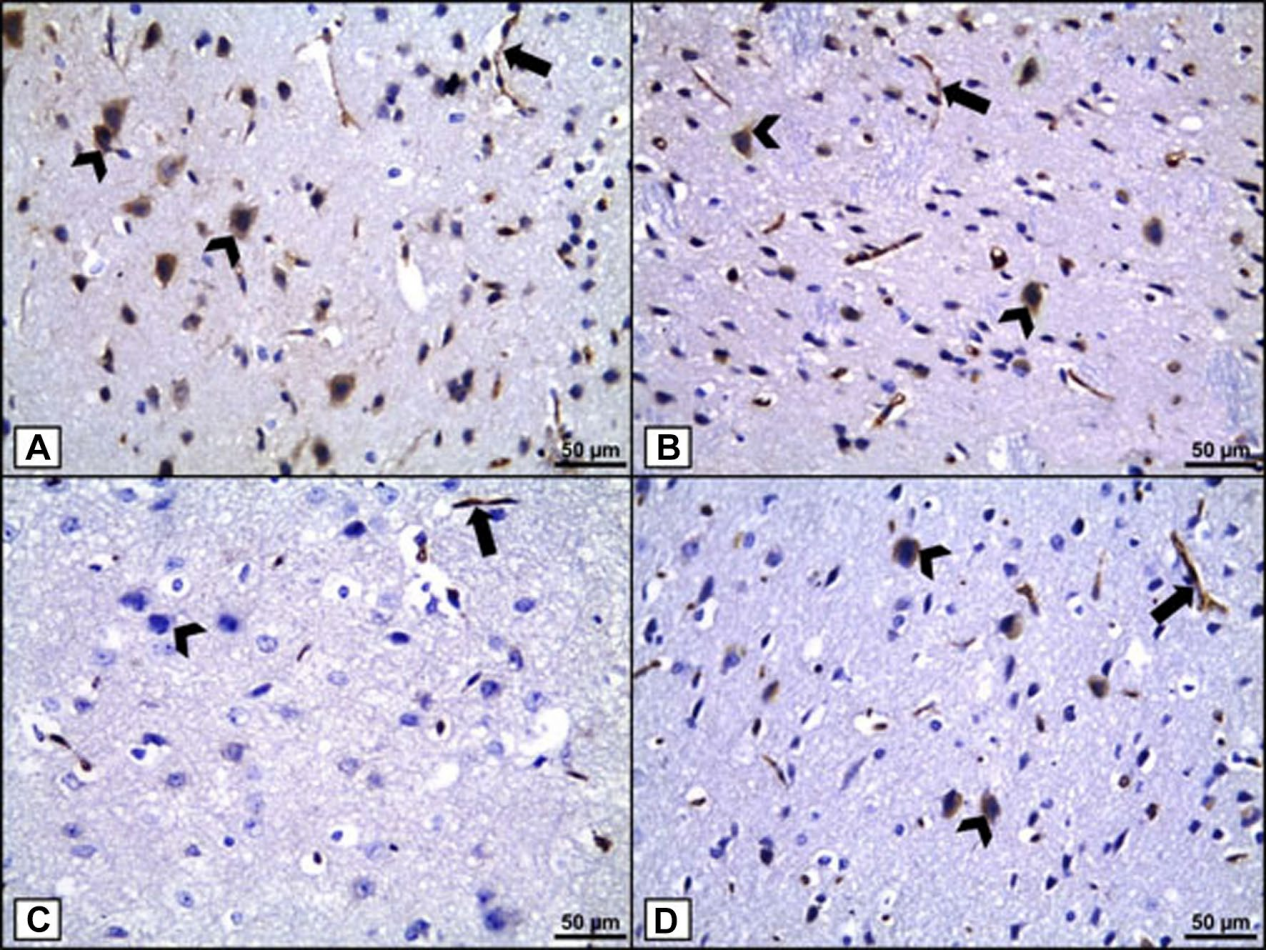
BCL-2 immunohistochemical staining. **A**, **B** Groups I and II show a strong positive cytoplasmic BCL-2 immunoreaction in numerous pyramidal cells (arrowheads) and blood vessel endothelium (thick arrow). **C** Group III shows a very faint cytoplasmic BCL-2 immunoreaction in most pyramidal cells (arrowhead), while blood vessel endothelium shows a moderate immunoreaction (thick arrow). **D** Group IV shows a moderate cytoplasmic BCL-2 immunoreaction in many pyramidal cells (arrowheads) and blood vessel endothelium (thick arrow). [Magnifications: (a-d) x400, scale bar = 50 μm]

In Groups I and II, IHC-stained sections for BCL-2 detection revealed a strong cytoplasmic immunoreaction in numerous pyramidal cells and the blood vessel endothelium (Fig. 6A, B). Moreover, sections of Group III revealed a very faint cytoplasmic immunoreaction in most pyramidal cells, while blood vessel endothelium showed a moderate immunoreaction (Fig. 6C). Additionally, sections of Group IV revealed a moderate cytoplasmic immunoreaction in many pyramidal cells and blood vessel endothelium (Fig. 6D). These findings suggest that alcohol can promote neuronal cell death, which can be prevented by NAG co-administration.

**Fig. 7:**
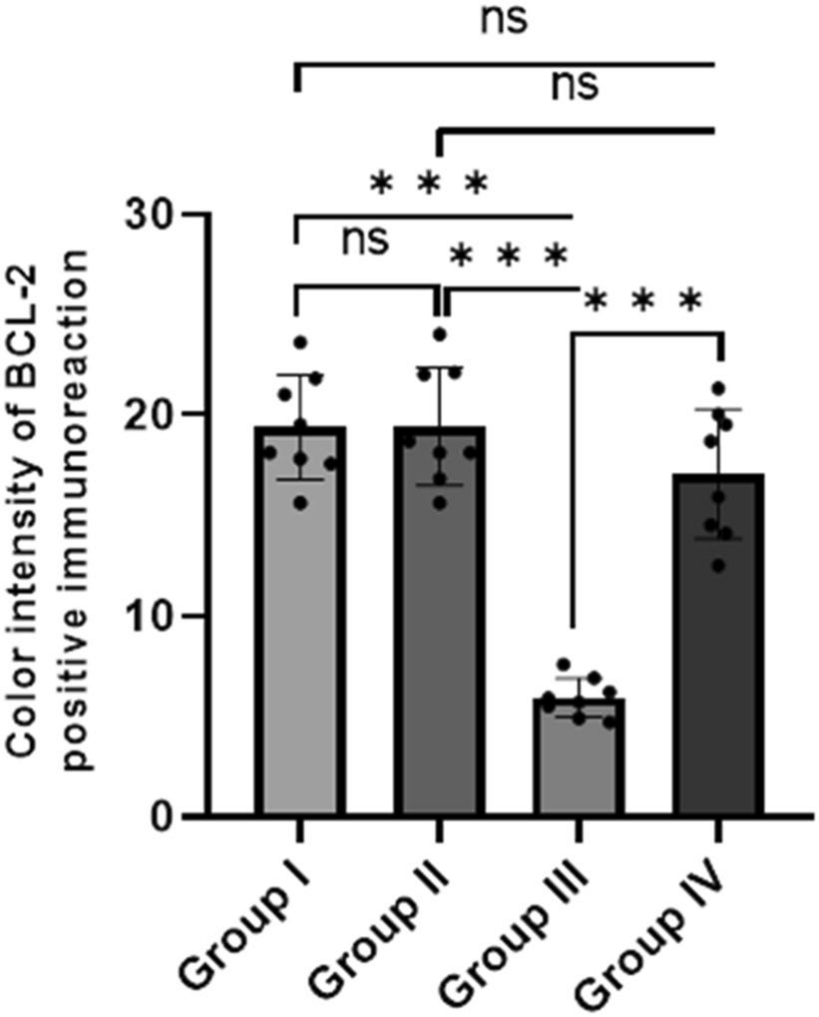
Mean color intensity BCL-2 positive immunoreaction of different studied groups. The values are represented as the means and standard deviations of eight rats in each group. ns: non-significant, *p < 0.05, **p < 0.01, and ***p < 0.001

A morphometrical analysis revealed that the mean color intensity of BCL-2 positive IHC reactions was significantly lower in Group III (5.98 ± 0.86) than in the control group (19.37 ± 2.19), but significantly higher in Group IV (17.09 ± 3.11) than in Group III (Fig. 7).

## Discussion

Alcohol has a high potential for abuse due to its sedative effect. Chronic alcohol consumption, as a life-threatening neurotoxin, has been proven to be associated with detrimental molecular and functional neuronal homeostasis, with great susceptibility to brain damage and behavioral and cognitive disabilities [25].

Despite significant progress in understanding its underlying pathophysiology, the molecular mechanisms of alcohol-induced neurodegeneration are far more complex. Furthermore, the potential contribution of the newly discovered necroptotic cell death is still unclear, and the possible protective effect of the natural citrus flavonoid, NAG, against alcoholinduced neurodegeneration has yet to be investigated.

Several mechanisms, including oxidative stress and neuroinflammation, have been implicated in alcohol-induced brain insults [26]. Growing evidence suggests that alcohol-induced brain injury is caused in part by a disrupted redox status caused by excessive production of reactive oxygen species (ROS) that is unbalanced by a compromised antioxidant system, resulting in oxidative stress. The brain, in particular, is highly susceptible to ROS effects due to its great oxygen needs and abundance of peroxidation-prone lipid membranes [27].

Alcohol-induced oxidative stress and mitochondrial dysfunction appear to be directly related to the excessive free radical and ROS formation in the brain, which leads to oxidative neuronal damage of cellular lipids and proteins and, eventually, neurodegeneration [28]. The current study confirmed NAG’s antioxidant capacity, as evidenced by increased GSH and decreased MDA brain levels. The antioxidant effect of NAG can be attributed to upregulated *Nrf2* expression and its downstream conserved target gene, *NQO-1*, which promotes NQO1 activity and raises GSH levels.

It has been reported that NAG acts on Nrf2 by downregulating Kelch-like ECH-associated protein 1 (Keap1), which acts as a cytoplasmic repressor of Nrf2 and is responsible for its ubiquitination and proteasomal degradation, keeping Nrf2 inactive under normal conditions [12]. Furthermore, in response to stress, *Nrf2* is released from Keap1 and translocated to the nucleus, where it increases the expression of antioxidant proteins such as NQO-1 and glutamate-cysteine ligase, the first enzyme in cellular glutathione biosynthesis. Therefore, molecules that target the Nrf2 pathway, such as NAG, may aid in developing novel treatment strategies for alcohol-induced oxidative stress-related disorders [29].

Alcohol-induced ROS could induce lipid peroxidation in cell membranes and other critical cellular macromolecules. NAG supplementation significantly reduced MDA levels in alcohol-treated rats, which could be attributed to its antioxidant and free radical scavenging action, as well as its polar nature, which facilitates adherence to the lipid bilayer and prevents peroxidation [30].

Neurodegenerative disorders are characterized by a neurotrophic factor deficit. CNTF is one of the most important neurotrophic factors involved in cell survival, and it has the potential to prevent neurodegenerative diseases [31]. Alcohol-induced oxidative stress may disrupt neurotrophic factors, which may be responsible for the neuronal damage observed in neurodegenerative diseases. The current study revealed that CNTF levels were significantly lower in Group III than in the other groups, while NAG co-treatment resulted in a significantly higher level. The increased level of CNTF in the NAG co-treated group can be attributed to its antioxidant properties and an up-regulated *Nrf2* signaling cascade, which tightly orchestrates the expression and activity levels of neurotrophic factors, as previously reported [12].

Indeed, it is well known that accumulating ROS, particularly mitochondrial ROS, is a crucial regulator of necroptosis [32]. Both RIPK3 and its downstream target, MLKL, are key players in this machinery, which is a cornerstone of neurodegenerative diseases. RIPK3/MLKL interaction, as previously reported by Zhang et al., causes MLKL activation, oligomerization, and translocation to the plasma membrane, resulting in cell necroptosis [33].

Necroptosis is thought to be crucial in the pathogenesis of a number of neurodegenerative diseases. Furthermore, necroptosis has received attention due to its potential as a therapeutic target in neurodegenerative disorders [34]. Our findings identified RIPK3/MLKL-mediated necroptosis as a crucial driver of neuron cell death in alcohol-induced neurodegeneration with the upregulation of necroptotic biomarkers. NAG treatment provided significant neuroprotection by suppressing the necroptotic pathway. Consistent with the current findings, Qian et al. [35] reported that RIPk3 could be upregulated via ROS production and that treatment with ROS scavengers had an anti-necroptotic role associated with oxidative stress inhibition, thereby protecting against cellular damage. Furthermore, Chtourou et al. [36] reported that NAG could inhibit necroptosis machinery in the heart in an experimentally induced hypercholesterolemia model.

Evidence suggests that chronic alcohol exposure makes cells more susceptible to apoptosis [37]. It has been reported that apoptosis is required for nervous system development and that, under various pathological conditions, upregulation of the death receptor family can sensitize nervous system cells to apoptosis as well as necroptosis, which is mediated by RIPK1, RIPK3, and MLKL [38].

The Bcl-2 family is a well-defined protein family involved in apoptosis that is tightly regulated by dynamic anti-apoptotic and pro-apoptotic interactions [39]. The current study found a significant reduction in the anti-apoptotic BCL-2 expression in Group III, whereas NAG co-administration significantly increased its expression, as previously reported by Al-Dosari [14] and Wang et al. [40]. In harmony with current findings, Shi et al. [41] found that BCL-2 could inhibit necroptosis effectors, MLKL alone, and the MLKL/RIPK3 complex, reducing their oligomerization and activation and thus inhibiting necroptosis and cell damage.

In line with our findings, Yuan et al. suggest that targeting RIPK1 may help inhibit apoptosis and necroptosis pathways, thereby ameliorating neuroinflammation [38]. At the same time, Hua et al. [42] reported NAG-mediated activation of the phosphoinositide 3-kinase (PI3K)/protein kinase B (Akt) pathway, most likely via suppression of phosphatase and tensin homolog (PTEN), a negative regulator of the PI3K/Akt/GSK-3β signaling pathway.

Previous studies have linked the PI3K/Akt/GSK-3β axis to neuronal survival, with activation possibly downregulating pro-apoptotic factors and upregulating anti-apoptotic proteins like BCL-2 proteins, thereby protecting cells from both apoptosis and necroptosis. GSK-3β is a key kinase in the phosphorylation of tau proteins, which is essential in neurodegenerative disorders. Its inactive form is phosphorylated [43]. NAG reduced pGSK-3β levels in Group IV, most likely by activating the PI3K/Akt pathway, and inactivated pGSK-3β could reduce tau toxicity, neuroinflammation, and neurodegeneration [43]. The current study demonstrated the neuroprotective effect of NAG co-administration in reducing pGSK-3β levels, as previously reported by Hua et al. [42] who identified NAG as a promising factor due to its protective effect against neurodegeneration.

According to Harper [44], alcohol may alter the brain both structurally and functionally by destroying brain cells and shrinking whole brain tissue by causing brain cells and connective tissue cells to expel water, resulting in structural and/or functional loss (i.e., neurodegeneration), as represented in our current study by a significant decrease in brain weight and volume and altered neurobehavioral tests of alcohol-intoxicated rats, with a significant improvement in the NAG-treated one. The findings presented here confirm those previously reported by Teixeira et al. [45]. Overall, the findings suggest that naringenin can help treat cognitive impairment by fighting neuroapoptosis, neuroinflammation, and oxidative stress.

There are some limitations to this study. First, the NAG effect on ameliorating alcohol-induced neurotoxicity has only been studied in the cerebral cortex, despite the fact that alcohol has a toxic effect on other regions of the brain, particularly the cerebellum, as well as other body organs such as the liver, which indirectly promote neurotoxicity. Second, no proteomic analysis has been performed to investigate the role of the ubiquitin-proteasome system and autophagy pathways in the elimination of malformed proteins. Therefore, additional research on other brain areas and organs, as well as proteomic analysis to identify cerebral protein alterations, is warranted to support our findings.

## Conclusion

The current study identified necroptosis as a major contributor to neuron cell death in alcohol-induced neurodegeneration using a well-established rat model. Interestingly, the current study uncovered that the natural citrus flavonoid, NAG, provides neuroprotection and behavioral recovery against alcohol-induced neurodegeneration, most likely through inducing *Nrf2* and its downstream signaling, therefore alleviating oxidative stress. Furthermore, NAG increased pro-survival/neuroprotective CNTF levels while suppressing RIPK3/MLKL-mediated necroptosis, which was mediated in part by a modulated GSK-3/BCL-2 axis.

The findings in this study suggest that NAG is a multi-target neuroprotective agent against alcohol-induced neurodegeneration via modulation of multiple cellular signaling cascades, making it a promising therapeutic target. Furthermore, additional clinical studies on NAG should be conducted to demonstrate its neuroprotective mechanisms in humans.

## Data Availability

The authors confirm that the data supporting the findings of this study are available within the article.
